# “The whole package deal”: experiences of overweight/obese women living with polycystic ovary syndrome

**DOI:** 10.1186/s12905-020-01090-7

**Published:** 2020-10-02

**Authors:** Carolyn Ee, Caroline Smith, Lisa Moran, Freya MacMillan, Michael Costello, Brandi Baylock, Helena Teede

**Affiliations:** 1 NICM Health Research Institute, Western Sydney University, Locked Bag 1797, Penrith, NSW 2751 Australia; 2 Graduate Research School, and NICM Health Research Institute, Western Sydney University, Locked Bag 1797, Penrith, NSW 2751 Australia; 3 Monash Centre for Health Research and Implementation - MCHRI, School of Public Health and Preventive Medicine, Monash University, Level 1, 43-51 Kanooka Grove, Clayton, Vic 3168 Australia; 4 School of Health Sciences, and the Translational Health Research Institute, Western Sydney University, Locked Bag 1797, Penrith, NSW 2751 Australia; 5 School of Women’s and Children’s Health, UNSW, Royal Hospital for Women, Barker St, Randwick, NSW 2031 Australia; 6 Western Sydney University, Locked Bag 1797, Penrith, NSW 2751 Australia; 7 Monash Centre for Health Research and Implementation – MCHRI, School of Public Health and Preventive Medicine, Monash University, Level 1, 43-51 Kanooka Grove, Clayton, Vic 3168 Australia

**Keywords:** Polycystic ovary syndrome, Obesity, Weight, Psychological

## Abstract

**Background:**

Polycystic Ovary Syndrome (PCOS) is a common female reproductive disorder with multiple manifestations. There are relatively few qualitative studies exploring the nature of living with PCOS despite its high prevalence. Qualitative research can enhance clinical practice via the provision of patient insights into the experience of living with their condition.

**Methods:**

We conducted two focus groups and three semi-structured interviews of Australian overweight/obese women with PCOS aged 18–46 years between March and April 2017 who were recruited through social media advertising. Interviews and focus groups were audio recorded and transcribed verbatim. Thematic analysis was applied to the data, using the method of constant comparison.

**Results:**

Ten women contributed data from two focus groups and two semi-structured interviews. Five themes emerged from the data: complexity of the condition with its multiple manifestations, difficulties with delayed diagnosis and lack of information provided after diagnosis, negative experiences on social media and online forums and the need for support, frustration over lack of a “cure”; and the impact of symptoms and concern about long-term sequelae.

**Conclusions:**

Living with PCOS appears to generate a significant degree of anxiety about the future, dissatisfaction with current treatment models, and loss of feminine identity. Gaps in timely diagnosis, information and support provision need to be addressed. This includes supporting weight management as a fundamental concern for women with PCOS.

## Background

Polycystic Ovary Syndrome (PCOS) is a complex hormonal disorder affecting up to 13% of women [1–4]. The manifestations of PCOS range from reproductive (oligomenorrhoea/amenorrhoea and chronic oligo/anovulation with resultant subfertility), hyperandrogenism (hirsutism, acne, alopecia), metabolic (dyslipidemia, insulin resistance) and psychological (mood disorders, body image disorders, and psychosexual dysfunction). Moreover, PCOS is a disorder that persists through the lifespan [1]. A meta-analysis has reported that women with PCOS have an increased prevalence of overweight [RR (95% CI): 1.95 (1.52, 2.50)], obesity [2.77 (1.88, 4.10)] and central obesity [1.73 (1.31, 2.30)] compared with women without PCOS [5]. Weight gain is a common presenting complaint [6]. Obesity has a negative impact on reproductive health [5, 7–9] and is associated with psychological co-morbidities [5, 10, 11].

There is some literature about women’s experiences of living with PCOS, which for many women includes lengthy delays in diagnosis and followed by inadequate or no provision of information at diagnosis [12, 13]. Following diagnosis, a range of both positive and negative reactions have been reported including shock, overwhelm, and anxiety over long-term risks [14]. Studies report that quality of life (QoL) is reduced in women with PCOS [15–20] compared to women without. Women with PCOS have expressed a number of concerns around fertility including whether they can fall pregnant, and what pre-conception preparation is needed [21]. However, there are relatively few qualitative studies exploring the nature of living with PCOS. Qualitative research is proposed to enhance clinical practice because it “generates a rich description of both local contexts and individual subjective experiences” [22]. Patient perspectives and insights into the subjective experience of living with a chronic condition may complement findings from quantitative research, highlight gaps in care, and help inform priorities for future research.

In this paper we report on the experiences of obese/overweight Australian women living with PCOS drawn from a qualitative study in order to inform and guide future patient-centred care for women with PCOS.

## Methods

This qualitative study is part of a larger study assessing the feasibility and acceptability of a method for a randomised controlled trial (RCT) on acupuncture and lifestyle for weight management in overweight/obese women with PCOS [23]. In the process of gathering data on the outcomes of interest for women, we elicited and explored women’s experiences of having PCOS. The objectives of this study were to report on the experiences of living with PCOS according to women in our study, in order to highlight gaps in care and inform the provision of patient-centred healthcare.

### Study design

We conducted a mix of both focus groups (two groups of five women and two women each) and semi-structured individual telephone interviews (three interviews), as not all women were able to attend the planned focus group sessions. We aimed to conduct focus groups of 90 min length and interviews of 30 min length. This study received ethical approval from Western Sydney University Human Research Ethics Committee (H11935/28 Nov 2016). All participants provided written informed consent.

### Participants

Women living in Sydney, Australia were recruited from the community through social media sources including paid advertisements on Facebook and posts on relevant women’s health organisation pages. We included women if they self-reported having been diagnosed with PCOS and self-reported having met the 2003 Rotterdam Criteria [16]; had a body mass index (BMI) > 25 kg/m^2^ determined from self-reported height and weight; were aged 18–46 (ie reproductive age); had no other endocrine disorders; were not pregnant or had no pregnancies in preceding 6 weeks; were fluent in English; and were able to attend for a focus group in the Western Sydney area. Women self-screened using an online questionnaire delivered using Qualtrics software [24].

We were interested in the views of women who were trying to conceive as compared with women who were not, as we hypothesised that the motivations for joining a clinical trial in these two groups may differ. As such we used purposive sampling to recruit women to two focus groups aiming for a sample size of 5–8 participants per group. We combined the focus group data with interview data in two groups according to pregnancy intention.

### Clinical and demographic variables

We collected basic demographic data including age, self-reported height and weight, ethnicity, educational level, employment status, and number of children.

### Data collection and analysis

An experienced research officer (BB, M.BioStats) facilitated the focus groups and two other researchers (CE, PhD and LI, undergraduate) observed and took field notes. Focus groups were held on a weekday evening, in a University setting while interviews were conducted over the telephone. All researchers are female. BB has a Health Psychology background, a broad range of experiences across health sciences but little prior knowledge of PCOS. CE is a medical doctor (general practitioner/family physician) and acupuncture researcher with a strong research interest in PCOS. LI is an undergraduate Health Promotion student with an interest in PCOS. CE and LI had some experience in qualitative research while BB had extensive experience. LI conducted the telephone interviews. The three interviews and two focus groups explored women’s experiences of living with PCOS, including motivation to enrol in the study, and clinical outcomes of interest, using a question guide. The question guide was not pilot tested. There were no relationships with participants that were established prior to study commencement. Participants were aware that CE is a GP and researcher with an interest in acupuncture for PCOS, and that BB and LI were research assistants. Women were told that the main research goal was to assess the feasibility and acceptability of a method for a clinical trial on acupuncture for weight loss in PCOS. No personal goals for conducting the research were divulged to participants and no non-participants were present. Repeat interviews were not carried out. Focus group discussions and telephone interviews were audio-recorded and professionally transcribed verbatim. Data was collected until data saturation was reached, which was determined by discussion between CE, LI and BB. Transcripts were circulated to participants for checking and no corrections were requested.

We used the method of constant comparison [25] in thematic analysis of the data [26]. The constant comparative method is an inductive data coding process that enables researchers to derive an understanding of phenomena through the everyday experiences, actions and words of participants, and provides a systematic method for organizing, comparing, and understanding similarities and differences between perceptions [27]. A subset of the transcripts was independently coded by three members of the research team (BB, CE, FM). Following this the team members agreed on a set of codes, and one investigator (BB) conducted a second reading of the codes and subcodes, and created a final set of emergent codes. These were then used to compare groups within the higher order themes. Approximately 20% of transcripts were cross-checked in this manner. We used Microsoft Excel 2016 version 15.40 to manage data. All women were given pseudonyms to protect their identities. None of the investigators were aware of any potential biases or assumptions about PCOS although CE was particularly interested in the role of complementary therapies for weight loss in PCOS. To ensure reliability and validity, we used the strategies of thick description, triangulation for reliability, peer review debriefing, developed a coding system, used member checks, and considered researcher bias [28].

## Results

Women were recruited from December 2016 until March 2017. There were 194 enquiries, of which thirty women were eligible. Of these, ten contributed qualitative data. The remaining women were uncontactable (9 women), were unable to attend focus groups due to distance and/or time constraints (8 women), or failed to attend focus groups and were subsequently uncontactable (3 women).

We ran two focus groups of two women and five women (not trying to conceive and trying to conceive respectively) in March and April 2017. Three women could not attend focus groups due to unforeseen circumstances on the day, and subsequently agreed to be interviewed. One was not trying to conceive, while the other two were trying to conceive. Interviews were conducted in April 2017. Focus groups lasted 104 and 117 min respectively, and the mean duration of telephone interviews was twenty-one minutes (range 17 to 27 min).

Of the ten women who contributed qualitative data, nine women completed the demographic survey, while one did not return the survey. Women were aged between 27 and 46 years old (mean age 43.5 years in the not TTC group and 34 in the TTC group) with a mean BMI of 36 kg/m^2^. Five out of ten women were married, nine worked full time, and six had at least a Bachelor degree qualification. Five women were of European ancestry, three were of Asian ancestry, and one of Oceanic ancestry. One woman had four children; the other respondents did not have any children. Five women in the TTC group reported difficulty in getting pregnant (no pregnancies despite TTC for 12 months) while the other two women had been TTC for less than 12 months.

### Themes

Five themes emerged on the experience of living with PCOS: the complexity of the condition; difficulties with diagnosis; negative online experiences and the need for support; frustration over lack of a “cure”; and the impact of symptoms and long-term sequelae. Additional quotes and sub-themes are provided in Table 1.

**Table 1:** Additional quotes

Theme	Subtheme	Quote
“The whole package deal”	Intrinsic, complex disorder	*“There’d be at least one in three women that have it and are dealing with it on a constant basis. Whether they find that through infertility or weight loss or whatever else other things. I think the down side of it is that it’s so intrinsic with the rest of your health. You actually don’t realise it until you’ve got it. Someone says, hey have a look at this do you know that this affects lots of things. Insulin resistance being the biggest factor of it. But it’s also intertwined and you don’t realise until you’ve actually been told” - Annabelle, not TTC*
	Lack of control	*“So I think the health benefits of it are that if you can get control of all of it then you end up with a better life I guess at the end. Because you’re not controlled by the PCOS and it’s not damaging the rest of the things that go wrong while you have it” – Annabelle, not TTC*
	Everything is linked	*“So the weight management and the fertility would be my top things. Because I think that if I maintained a healthy weight, if I got to a healthy weight and maintained it, I think that would help with the self-confidence and stuff” - Grace, TTC*
Diagnosis	Delayed diagnosis	*“So after that I found it’s hard to get pregnant. That’s when the doctor told me that you know that’s the reason and I took the pill for so long and all of that. Yeah, I wish someone had told me before” - Harriet, not TTC.* *“I know for me until I was diagnosed with PCOS trying to have my third child I didn’t know I had it at all. So I would have had no clue. But when I look at what it was and I’ve had irregular periods from the time I was 13 years of age and heavy periods not just normal periods and that’s what constituted the hysterectomy” - Annabelle, not TTC.*
	Lack of information after diagnosis	*“I went to the doctor and the doctor just gave me the tablets, the contraception, the pill. I was only 16. I’ve had it for years and I’m sick of it. Then I have a lot of acne. I have a lot. And hair. I got a lot of hair. Thank god for hair lasers. So all my facial hair is gone but I wasted a lot of money on it. But I hadn’t been told. I hadn’t been told for a long time. All I had been told was take the pill” – Annabelle, not TTC*
Negative experiences on online forums and social media, and the need for peer support	Negative experiences online	*“I generally skip over the positive ones just because I think good luck to them and all the best but that’s not me. I’m like you, that’s not happening to me so [I don’t like it]” Keira, TTC*
	Need for support	*“The support group. For me that would be something I would probably look forward to” – Chloe, TTC*
	Isolation	*“I would. I don’t know anyone else that has PCOS. Sometimes I’ll talk to my girlfriends and stuff and they just don’t get it. So it [a support group] would be good…” - Grace, TTC*
No cure	No cure	*“I personally, I find it interesting. With me, I would definitely give it a go. Because of the fact that I have tried so many other things and they’ve not worked, so I would 100% give it a goal. Other people, I reckon - I would imagine that there is a lot of other females that have PCOS that have had similar [unclear” – Grace, TTC*
	Altruism	*“So I guess having the incentive there to do a trial and a study, and also help the university out as well, so I guess, yeah, it’s a bit of an incentive to do it...A bit of a push, and there’s someone relying - not so much relying on me, but you get what I mean” – Georgia, not TTC*
PCOS symptoms and long term sequelae	PCOS symptoms	*“I get a lot of facial hair that I get really self-conscious about” - Grace, TTC* *“Now everything is like, I don’t know if it’s too personal or not, but even things such as hormone levels, sexual desire. All that sort of stuff I think has a lot to do with PCOS itself because you’re not firing on all eight cylinders like you should be” - Annabelle, not TTC* *“I have pretty irregular periods and pretty emotional too; like I guess just the mental health [outcomes] as well - I’m very hormonal, so probably look at that” - Georgia, not TTC* *“Yeah, I think that the depression and how you feeling mentally about it they should because you said we get anxiety and things like that” - Jessica, TTC.* *“Because I suffer with depression a lot and I was told that that can sometimes go hand-in-hand with having the PCOS. So, dealing with the self-esteem side of it, that would be a big thing for me” - Grace, TTC.* *“I think at the moment, my top two priorities would be the healthy weight and then dealing with the fertility...So the weight management and the fertility would be my top things. Because I think that if I maintained a healthy weight, if I got to a healthy weight and maintained it, I think that would help with the self-confidence and stuff” - Grace, TTC*
	Chronic disease risk	*“But I think the blood pressure, yeah, the heart and the anxiety is really controlling and I know, yeah, that’s my problem” – Harriet, not TTC*
	Psychological aspects	*“Because I suffer with depression a lot and I was told that that can sometimes go hand-in-hand with having the PCOS. So dealing with the self-esteem side of it, that would be a big thing for me” - Grace, TTC*

*PCOS* Polycystic Ovary Syndrome, *TTC* Trying To Conceive

#### (1) “The whole package deal“

A theme that emerged from all women was the complex nature of PCOS, referred to as the “*whole package deal*”. Women were well aware of the multiple manifestations of PCOS, and spoke of their desire to improve all aspects of PCOS including weight, menstrual cycles, hirsutism and acne, and psychological aspects. They felt a lack of control from the internal and external manifestations of their condition. Additionally, they knew that they would have some manifestations of PCOS for the rest of their life.

*"All that packaging that comes with PCOS - hormone imbalance and [unclear], facial hair and all that other stuff [unclear] whatever is happening inside your body because you know something's not right there - so the whole package deal I guess, because it does lead to problems "* - Keira, TTC

Many women understood that all aspects of PCOS were linked, and if one aspect improved (such as weight), others would as well. Georgia said, when asked about her top priority:

*"Probably maintaining a healthy weight, because with a healthy weight comes regular periods and a better mental health"* - Georgia, not TTC.

#### (2) Diagnosis

Some women had experienced lengthy delays before being officially diagnosed with PCOS.

*"I went to the doctor and the doctor just gave me the tablets, the contraception, the pill. I was only 16. I’ve had it for years and I'm sick of it. Then I have a lot of acne. I have a lot. And hair. I got a lot of hair. Thank god for hair lasers. So, all my facial hair is gone but I wasted a lot of money on it. But I hadn't been told. I hadn't been told for a long time. All I had been told was take the pill" -* Harriet, not TTC.

Even after diagnosis, very little information was initially given on the cause, complications and management of PCOS.

*"I was diagnosed with PCOS 17 years ago and even then, it wasn’t very well talked about. It wasn’t very well known. The doctor just basically said, oh you've got PCOS. I went okay thanks what does that mean and not much information was given" -* Annabelle, not TTC.

#### (3) Negative experiences on online forums and social media, and the need for peer support

Some women, particularly those who were trying to conceive, spoke of negative reactions to positive posts on online forums and “Facebook” pages, which were usually about In Vitro Fertilisation (IVF) or fertility in general. Learning about other women falling pregnant could provoke anxiety or a sense of isolation.

*"I think what happens is sometimes - it depends when I'm - what time it is at night or day. If I go on and someone's posted something and I go I can relate to that, if I click in and see comments I just start to get anxious and I'm like well why is Clomid working for me. She only took it three times and now she's pregnant" -* Chloe, TTC

Online forums, despite the negative reactions described, could sometimes offer women the ability to find women who were going through the same struggles, and whom they could relate to. Women were generally not getting this support from their own social group and this could lead to a sense of isolation.

*"Maybe you'll find two or three people who are like yeah I feel the same." -* Keira, TTC.

*"I don't know anyone else that has PCOS. Sometimes I'll talk to my girlfriends and stuff and they just don't get it. So, it would be good…[to have support from other women with PCOS]" -* Grace, TTC.

#### (4) “Still now in 2017 we do not have a cure for this”

In the process of contributing data on the feasibility of the clinical trial, all women spoke of altruistic reasons for taking part in the study. There was a sense of the need to find a cure for PCOS. Women in the TTC group spoke of the frustration they felt at the paucity of acceptable treatments for PCOS, despite it being such a common condition.

*"This is not a very rare condition or anything. You would see every other woman nowadays have PCOS. It just feels bad and something that we're missing that we do not have a cure for this. Still now in 2017 and we don't have a cure for this, it's like something so rare. "* ***-*** Keira, TTC.

Women who were not TTC spoke similarly in terms of contributing to research and to prevent the younger generations from suffering in the way that they had. In particular, Annabelle spoke of her concern for her four daughters:

*"I look at something like this as being it's not just about me at the end of the day. If it means it helps somebody else in the study and that’s the main aim of it is to be able to help other people to not have to struggle as much. I mean I see that at home with four girls. But as I said it's so prevalent now if there is a way to assist anybody to get what they want. Whether that’s a baby, a better lifestyle, weight loss"* - Annabelle, not TTC.

#### (5) PCOS symptoms and long term sequelae

A consistent theme across all participants was the long struggle they had had with losing weight and the number of interventions they had tried. Annabelle (not TTC) had been trying for 27 years; Harriet (not TTC) since she was a teenager; Jessica (TTC) described “*a lifetime of yoyo dieting*”.

*"So you change it for a while and you're all good and then you sort of go back to your normal eating and your normal pattern. Then you just go up and down. So you're always doing the up and down stuff"-* Annabelle, not TTC

Some women found the concept of a hormonal imbalance quite distressing, as well as coping with the external manifestations of hyperandrogenism, and the impact on libido.

*"Hormone balance as well because I was pretty shocked when my doctor said that you have a high level of [testosterone]. I was like oh my god I'm turning into a [man] somehow" -* Keira, TTC.

A number of women spoke about battling depression, anxiety and mental health issues. Low self-esteem from being overweight and having acne and facial hair were contributors to poor mental health for some women, as was anxiety about having a sudden and unexpected menstrual bleed. There was an understanding of the link between psychological symptoms and PCOS.

*"It can make you more anxious about the situation you're in. You know back when I did have periods things of just having the irregularity of them and not knowing. Hey, they're due round about now and so preparing yourself to go hey I better put some things in my handbag. Or I know they're coming so I know they are going to arrive on this day. Or I'm not going to bleed through when I stand up" -* Annabelle, not TTC.

Women in both groups were aware of the long-term sequelae of PCOS, and spoke about their fears of developing diabetes and heart disease in the future. However, the women who were not TTC spoke more frequently about their concerns of impending diabetes. There was a sense of the continuity of PCOS and how it affected each life stage from menstruation, fertility, to long term health. Controlling weight was seen as pivotal as it could impact on all aspects of PCOS - it could improve fertility, outward appearances (weight and hyperandrogenism), self-esteem and mental health, and risk of chronic disease. It offered women a sense of hope. Annabelle summarised it as follows:

*"So, I think the health benefits of it are that if you can get control of all of it then you end up with a better life I guess at the end. Because you're not controlled by the PCOS and it's not damaging the rest of the things that go wrong while you have it. I don't think they're exclusive. I think it's a package deal that if you certainly can control the weight - control the PCOS that helps to control the weight then health benefits are just continuous"* - Annabelle, not TTC.

## Discussion

This paper reports on the challenging experience of living with PCOS. Women in our study struggled with the complex nature of PCOS, consistent with that found in other qualitative studies. Crete et al. conducted semi structured interviews with ten women with PCOS, reporting four major themes (frustration, confusion, searching, and gaining control) [29]. In another qualitative study, a major theme was the ongoing struggle to control symptoms, particularly weight [30]. There were similar themes of concern for older self, especially with regard to future infertility; feeling physically inferior; coming to terms with a chronic condition; shock at the complex nature of the condition with multiple health implications in the future [30]. Future research on women with PCOS should capture the varied aspects of this disorder, such as mental health outcome measures, in order to evaluate changes that are meaningful to women. Clinicians should be aware of the dynamic and diverse nature of PCOS throughout the reproductive lifespan and beyond, the impact on women’s quality of life, and the need to screen for cardiovascular risk factors and be aware of associated disorders such as obstructive sleep apnoea and endometrial cancer. Our findings support the recommendations in evidence-based guidelines on PCOS to utilise a multidisciplinary approach [31].

Women in our study expressed frustration at lengthy delays in diagnosis, and not being given adequate information after diagnosis, consistent with previous research [13, 32–34]. There are gaps that need to be addressed with timely diagnosis, providing comprehensive, specific and practical health information, and adequate training of health practitioners.

Williams et al. found that women in one study reported that PCOS had affected their feminine identity, threatening their identity as a woman [35] which was echoed by women like Keira in our study, who said “*Oh my god I’m turning into a man somehow*”. Similarly, in another qualitative study, some women were distressed by hirsutism which made them feel “*like a freak*” [36]. Quantitative studies have reported that the physical symptoms of PCOS may cause a loss of feminine identity, a feeling of being unattractive, and reduced QoL, although studies in this area are limited [18, 37]. Physical features of PCOS such as hirsutism and increased weight appear to impact negatively on body image and QoL [38, 39] which may then be associated with depression [40, 41]. Annabelle alluded to sexual dysfunction and “*not firing on all eight cylinders like you should be”.* The prevalence of sexual dysfunction in women with PCOS ranges from 13 to 62% and appears to be greater than in women in the general population [37]. Therefore, as per the new international evidence-based guidelines on PCOS, clinicians and health professionals caring for women with PCOS should be aware of the increased prevalence of psychosexual dysfunction, reduced QoL, and body image distress in this population, particularly if there are features of hirsutism, overweight/obesity, and should screen, assess and refer women for support and treatment as appropriate [31].

Some women in our study reported battling anxiety and depression, and most of all low self-esteem. Similarly, in the study by Williams et al., participants reported episodes of depression, self-harm and suicidal ideation [35]. Meta-analyses have consistently reported increased depression and anxiety scores in women with PCOS compared to women without PCOS [4, 42–44], although certainty of the evidence is somewhat limited by small sample sizes and heterogeneity. Health professionals who care for women with PCOS should be aware of the increased prevalence of mental health disorders in this population, and routinely screen for anxiety and depression in all women at diagnosis [31].

Based on our findings there appears to be a need for additional support from friends, health practitioners, and other women with PCOS. This is consistent with themes emerging from other studies identifying an unmet need for support from other women with PCOS [30], and reporting frustration from the lack of support from healthcare professionals, and active seeking of support on the internet including from other women with PCOS [35]. Women in our study were generally unaware of existing consumer organisations such as the Polycystic Ovary Syndrome Association of Australia, and voiced a need for PCOS-specific services. A range of supports appear to be necessary, such as PCOS-specific online forums, PCOS support groups (such as PCOS Challenge in the USA, and Verity in the United Kingdom), and access to educational materials for family members (spouses and children). Research on support groups as models of care for women with PCOS is limited, although two studies report some positive benefits from on-line [45] and nurse-led support groups [46]. Future research could explore the role of support groups in the management of PCOS.

Last, while women expressed their frustration at many aspects of living with PCOS, losing weight offered them hope, a way to improve multiple symptoms and minimise long term sequelae. Yet, all had struggled with “*a lifetime of yoyo dieting”.* Women with PCOS are more likely to be obese/overweight than age-matched controls [47], and weight management is a key goal in PCOS [48]. Excess weight worsens the features of PCOS [49] while weight loss improves its features [6]. The women in our study were aware that their primary goal should be weight loss, however all had struggled with weight loss and maintenance. There is a gap between what is known about the benefits of weight loss in this group, and the provision of effective, sustainable interventions for weight loss in PCOS.

This study has some limitations. The major limitation is that the sample size is small, with a relatively high dropout rate on the day of the focus group for the women who were not TTC. Therefore, themes may not be representative of the views of all women with PCOS or women with other demographic characteristics or living in other geographic locations. Specifically, we could not capture the experiences of women who could not speak English, although our sample is culturally diverse, with 30% of women being of Asian origin. However, we recruited until saturation was met in the main themes from the combined interview and focus group data, confirming the adequacy of the data that was collected.

We initially chose to conduct focus groups as this methodology can assist with reaching consensus between participants who have similar characteristics, and data is enriched by the interaction between participants [50]. To overcome pragmatic issues around inability to attend for face to face focus groups, we offered women the opportunity to be interviewed over the telephone. The themes identified across interviews and focus groups in our study were consistent, indicating that similar data were collected regardless of the method used.

## Conclusion

To the best of our knowledge this is the first qualitative study to explore Australian women’s experiences of living with PCOS. Consistent with similar qualitative studies internationally, we report a high level of concern and frustration amongst Australian women with PCOS around the complexity of the disorder, the impending burden of chronic disease, and low self esteem and mental health concerns relating to physical appearance and infertility. Living with PCOS appears to generate a significant degree of anxiety about the future, dissatisfaction with current treatment models, and loss of feminine identity. In order to improve the experience of living with PCOS, gaps in timely diagnosis and information and support provision need to be addressed. This includes supporting weight management as a fundamental concern for women with PCOS.

## Supplementary information

**Additional file 1.** Focus group/interview question guide.

## Data Availability

The datasets used and/or analysed during the current study are available from the corresponding author on reasonable request.

## Abbreviations

PCOS: Polycystic Ovary Syndrome
TTC: Trying To Conceive
RCT: Randomised controlled trial
