# Supplementary material for: “The whole package deal”: experiences of overweight/obese women living with polycystic ovary syndrome
Source: BMC Womens Health. 2020 Oct 2;20:221. doi: 10.1186/s12905-020-01090-7 (PMC7532653; doi:10.1186/s12905-020-01090-7)
Supplement: Supplementary file 1 — Additional file 1. Focus group/interview question guide. [file 12905_2020_1090_MOESM1_ESM.docx]

**Additional File 2: Focus group/interview question guide**

The facilitator/interviewer will ask the group/participant the following questions:

1. Past weight loss attempts
   1. Can you tell me about your previous attempts to lose weight?
   2. What weight loss approaches are you aware of? What do you think of them?
   3. What weight loss approaches have you tried? Why? How effective do you think they were? What was good about that approach? What wasn’t so good?
   4. What was it about that approach that you found helpful? What stopped you from trying a certain approach or got in the way of maintaining it?
2. acupuncture
   1. Have you heard of acupuncture? What do you know about it?
   2. Have you had acupuncture before?
   3. What do you think the disadvantages and advantages may be of using acupuncture in general?
   4. What do you think about using acupuncture as a weight loss treatment in conjunction with other treatments like lifestyle changes?
3. Interventions that will be delivered in the RCT. A verbal and written explanation will be provided: “We are planning a clinical trial that will compare acupuncture with placebo acupuncture for weight loss. Women will attend for 12 treatments of acupuncture over 12 weeks. They will attend twice weekly for the first 4 weeks and then fortnightly for 4 sessions. The acupuncture treatments will take 45 minutes at a time. All women who take part in this trial will also receive lifestyle coaching over the telephone, will set their own diet and exercise goals, will be encouraged to follow the national guidelines for diet and exercise that is a healthy balanced diet, and 2.5 hours of aerobic exercise a week. Women will receive ten 15-minute phone calls over 6 months. Women will attend for a clinic visit at Western Sydney University, Campbelltown campus, before and after the course of acupuncture treatment, and will complete some surveys about their health, as well as do some blood tests before and after the course of acupuncture treatment.”
   1. What do you think about this trial in general?
   2. Would you volunteer for a trial like this? Why? Why not? What would make it more appealing for you? What would make it more acceptable? What would motivate you to volunteer for such as trial?
   3. What do you think about the lifestyle coaching service that we will offer during this trial? What do you think of the time commitment? What do you think about the fact that it is being delivered over the telephone?
   4. What do you think about the acupuncture treatment frequency (ie twice a week for 4 weeks, then fortnightly afterwards)? Would you attend? What would motivate you to attend? What do you think an acceptable frequency of treatment would be? What would make it easier or harder for you to attend for all the treatments?
4. the control method of sham acupuncture and perceptions on how this might impact on recruitment and attrition, whether other attention control methods may be more acceptable such as massage or progressive muscle relaxation
   1. We are using placebo or sham needling in this study, where the needles are not inserted in the same way as done usually, and this is believed to render the treatment less active than real acupuncture. Women enrolling in our study will have a 50% chance of being allocated to real or sham acupuncture and will not know which group they are in. What do you think of this? Would it make you more or less likely to want to enrol in the study? Why? Why not?
   2. If we offered another type of control treatment, such as massage treatment or progressive muscle relaxation, would this make you more likely to volunteer than if the study involved only acupuncture or sham acupuncture? What are the advantages and disadvantages that you can see?
   3. What other control treatments would you like to see in these kinds of trials?
5. The outcomes important to them as end-users, the acceptability of the proposed outcomes in the RCT
   1. Apart from weight loss, what else is important to you in terms of improving your health with regards to PCOS? (e.g. fertility, menstrual cycles, risk of diabetes). Why is this important to you? Are these more or less important to you than maintaining a healthy weight? Why? Why not?
   2. In the trial, we are asking women to attend for a 2 hour Oral Glucose Tolerance Test before and after treatment. This test will involve at least 2 hours of your time, three blood tests over the two hours, and drinking a sugary drink after the first test. You will also need to fast overnight. It tells us about your risk of developing diabetes. What do you think of this test? Have you had it before? Is it important to you to know about your risk of diabetes? Would you enrol in the study if you knew you had to do these tests? Why? Why not?
   3. Are there any other outcomes that are important to you that you think we should measure in the study? (For example, quality of life, anxiety and depression).
   4. (in women who are planning to conceive) If you were asked to volunteer for the trial prior to trying to conceive, would this be acceptable to you? Would you wait until you had completed the trial before trying to conceive again?
6. Thinking about the study, what do you think the main barriers are in terms of getting women to enrol in it, and getting them to complete all treatments and tests? What do you think is helpful about the study design? What would help keep women in the study? Are financial incentives important? Where do you think we should advertise for volunteers for the study? Is there anything we should change to make it more acceptable and attractive to women with PCOS?
